# Utilization of Cannabidiol in Post-Organ-Transplant Care

**DOI:** 10.3390/ijms26020699

**Published:** 2025-01-15

**Authors:** Sachiko Koyama, Jumar Etkins, Joshua Jun, Matthew Miller, Gerald C. So, Debora L. Gisch, Michael T. Eadon

**Affiliations:** 1 Department of Medicine, Indiana University School of Medicine, Indianapolis, IN 46202, USA; jetkins@iu.edu (J.E.); joshjun@iu.edu (J.J.); dgisch@iu.edu (D.L.G.); meadon@iu.edu (M.T.E.); 2 College of Human Ecology, Cornell University, Ithaca, NY 14850, USA; mm2777@cornell.edu

**Keywords:** cannabidiol, organ transplant, inflammation, cytochrome P450, drug–drug interaction, pharmacokinetics, pharmacodynamics, adverse events, chemical formulation, cannabis plant chemical constituent

## Abstract

Cannabidiol (CBD) is one of the major phytochemical constituents of cannabis, *Cannabis sativa*, widely recognized for its therapeutic potential. While cannabis has been utilized for medicinal purposes since ancient times, its psychoactive and addictive properties led to its prohibition in 1937, with only the medical use being reauthorized in 1998. Unlike tetrahydrocannabinol (THC), CBD lacks psychoactive and addictive properties, yet the name that suggests its association with cannabis has significantly contributed to its public visibility. CBD exhibits diverse pharmacological properties, most notably anti-inflammatory effects. Additionally, it interacts with key drug-metabolizing enzyme families, including cytochrome P450 (CYP) and uridine 5′-diphospho-glucuronosyltransferase (UGT), which mediate phase I and phase II metabolism, respectively. By binding to these enzymes, CBD can inhibit the metabolism of co-administered drugs, which can potentially enhance their toxicity or therapeutic effects. Mild to moderate adverse events associated with CBD use have been reported. Advances in chemical formulation techniques have recently enabled strategies to minimize these effects. This review provides an overview of CBD, covering its historical background, recent clinical trials, adverse event profiles, and interactions with molecular targets such as receptors, channels, and enzymes. We particularly emphasize the mechanisms underlying its anti-inflammatory effects and interaction with drugs relevant to organ transplantation. Finally, we explore recent progress in the chemical formulation of CBD in order to enhance its bioavailability, which will enable decreasing the dose to use and increase its safety and efficacy.

## 1. Introduction

Patients who undergo organ transplantation often face chronic pain [1] for similar reasons as the general population. However, the options to alleviate pain in allograft recipients are frequently limited. The use of non-steroidal anti-inflammatory drugs (NSAIDs) is often contraindicated by concomitant kidney or cardiovascular disease. Opioid use is restricted to acute pain indications due to its addictive potential. Additional options to control pain in transplant recipients are needed.

Despite advancements in immunosuppressive medications and our understanding of human leukocyte antigen (HLA) types that differentiate self from non-self [2], we are still in need of agents that work to suppress chronic pain and improve the quality of life (QoL) of these patients. One of the most commonly used immunosuppressive agents is tacrolimus (FK506; PubChem ID 445643) [3,4], which prevents organ rejection and modulates immune responses. Tacrolimus functions by binding to the FK506-binding protein (FKBP), forming a complex that inhibits calcineurin. Calcineurin is a protein critical in the T cell proliferation signaling pathway. Detection of a foreign substance (antigen) by the T cell receptors (TCRs) triggers calcium influx through the calcium-release-activated calcium (CRAC) channel, leading to calcineurin activation. Activated calcineurin facilitates translocation of the transcription factor family, a nuclear-factor-of-activated-T-cells (NFAT), to the nucleus, initiating the T-cell activation and differentiation [5]. Thus, by inhibiting calcineurin, tacrolimus can effectively suppress T cell activation and proliferation [6]. 

The effects of tacrolimus can be influenced by various factors, particularly its metabolism. Tacrolimus, like many drugs and xenobiotics, is metabolized in the liver through the actions of two major enzyme families. Cytochrome P450 (CYP) and uridine 5′-diphospho-glucuronosyltransferase (UDP-glucuronosyltransferaze, or UGT) are the two major families of enzymes involved in drug metabolism: CYP at phase I and UGT at phase II [7]. Recent studies have found that there are polymorphisms in these enzymes, resulting in differences in tacrolimus metabolism depending on the subtype present [8,9,10]. These studies suggest that, following the practice of ‘precision medicine’, it is necessary to consider the genotypes of the patients to determine the appropriate dose of tacrolimus. Metabolic phenoconversion for drug–drug interactions (DDIs) is equally important, and several phytochemicals are known to interact with and modulate the activity of both CYP and UGT enzymes. Phytochemical compounds are one of the agents that provide this function by binding to CYPs and UGTs. 

Cannabidiol (CBD) is one of the most well-known phytochemical compounds and is metabolized by CYP and UGT [11,12,13]. It is commonly used as a supplement to suppress anxiety and has been incorporated into medical treatments for conditions such as epilepsy, cancer, and Parkinson’s disease [14]. CBD is also well-known as an anti-inflammation and antioxidant agent [15,16]. The image of CBD is that it is one of the major chemical constituents of cannabis. There are many other phytochemicals such as curcumin and piperine. Curcumin and piperine are major chemical constituents of turmeric and black pepper, respectively. They are also known to inhibit CYPs and UGTs [17,18,19] as strongly as CBD does (there are differences in which CYPs and UGTs they inhibit more. In a study by Shamsi et al. [20], which did not compare curcumin and piperine with CBD, they compared curcumin, piperine, and capsaicin and reported that piperine inhibited CYP3A4 and CYP1A2 at lower concentration (IC50 for CYP3A4 and CYP1A2 was 2.12 µM and 2.14 µM, respectively, for piperine, whereas it was 11.93 µM and over 100 µM for curcumin). Yamaori et al. [21] reported the IC50 of CYP3A4 was 11.7 µM, and Bansal et al. [22] reported the IC50 of CYP1A2 by CBD as 0.45 µM. Thus, for CYP3A4, the order of potency becomes piperine > CBD = curcumin and for CYP1A2, the order becomes CBD > piperine > curcumin. These studies indicate that the potency of inhibition varies depending on the types of CYP. These differences depending on the types also exist in the inhibition of UGT as well [12]), and yet not known to the general public compared to CBD. The psychoactive property and the addiction potential of cannabis [23] have been known for centuries, and the possession of the plant became federally regulated in 1937 in the U.S. 

In this review, we will summarize the historical background of CBD, the effects of CBD on drug metabolism, and the anti-inflammatory effects of CBD. We will discuss the adverse events reported in clinical trials using CBD and the potential strategies to mitigate side effects. The goal of this review is to explore the possibility of using CBD to improve the QoL of post-organ-transplant patients.

## 2. Background

### 2.1. Cannabis sativa, L.

Cannabis has been cultivated since ancient times [24]. Historically, the stems have been used to make cloth and the seeds used as food, in which the concentrations of chemical constituents that have psychoactive properties are low [24]. The chemical constituents with psychoactive properties of the plant are concentrated in the leaves and flowers/flower buds, with differences in these concentrations depending on the subspecies of the plant and among the wild/domesticated varieties of cannabis plants [25,26]. The subspecies with lower psychoactive properties (less than 0.2%) [27,28] is referred to as hemp (*Cannabis sativa* subsp. sativa). Cannabis is dioecious, exhibiting significant differences in the morphology, seed formation [26,29] and the concentration of the chemical constituents that cause psychoactive properties (female plants > male plants) [30]. All of these differences depending on the parts of the plant, sex of the plant, sub-species types, and wild/domesticated varieties have historically influenced its use across multiple domains. The plant has been used in various medical practices beginning in ancient times. Historically, it has been used as an anesthetizing agent and to treat rheumatic pain, gastrointestinal symptoms, and female reproductive system disorders [31]. Utilization of cannabis for medical practices is considered to date back to about 12,000 years ago [32], suggesting that the use of their stems to produce fabric and their seeds as food may have evolved in parallel to the use of leaves and flowers for medical practices. The psychoactive nature and medicinal use might have both been utilized in religious activities in ancient times and later, in more recent centuries, became used for recreation, with the growing additional understanding of the addictive properties along the time course. As mentioned above, the psychoactive properties and addiction potential started the movement to regulate the plant, which became a national regulation in the U.S. in 1937. This regulation limited use for medical treatments as well, but later, in 1997, use of cannabis was legalized for medicinal use in California. Legalization is now gradually expanding to other states. 

An important landmark for the phytochemicals contained in cannabis was the identification of cannabinoid receptors rather than the identification of the chemical constituents of cannabis. The findings of the chemical constituents of cannabis started as early as the end of the 19th century (cannabinol, CBN) [33,34], followed by other chemical constituents of cannabis including CBD in the 20th and 21st centuries. The terpenophenolic constituents of cannabis were named cannabinoids [35], and it was also determined that Δ^9^-tetrahydrocannabinol (THC) is the major chemical constituent involved in producing the psychoactive properties and addiction potential [36]. Importantly, in the 1980s, two types of cannabinoid receptors, cannabinoid receptor 1 (CB1, coded for by the CNR1 gene) and cannabinoid receptor 2 (CB2, coded for by the CNR2 gene), were found [33], and activation of CB1 was found to be involved in the psychoactive responses. CB1 was expressed mostly in the central nervous system, whereas CB2 was expressed mostly in the peripheral tissues and immune cells, and some were detected in the central nervous system [37]. The finding that CB1 was responsible for the psychoactive properties may have generated studies focusing on the central nervous system and delayed the progress of studies on other receptors, channels, and enzymes that cannabinoids can bind, such as transient receptor potential vanilloid 1 (TRPV1), peroxisome proliferator-activated receptor (PPAR)γ, and cytochrome P450 (CYP) [38]. In 1992 and 1995, two endogenous molecules, N-arachidonoylethanolamine (anandamide or AEA) and 2-arachidonoyl glycerol (2-AG), were found to activate cannabinoid receptors [39,40] and so were called endocannabinoids. It was also found that there are non-cannabinoid chemicals included in cannabis and in other plants that activate the cannabinoid receptors, and thus the term phytocannabinoids was coined as “any plant-derived natural product capable of either directly interacting with cannabinoid receptors or sharing chemical similarity with cannabinoids or both” [35]. 

It is easy to imagine that this extensive historical background on cannabis and cannabinoids had profound influences in a number of ways on the studies of CBD. First, when CBD was found to have anti-anxiety and anti-inflammatory effects without psychoactive influences, this made CBD a strong candidate to become a drug for anxiety and inflammatory symptoms, although there are many other symptoms that could be treated with CBD. Studies are catching up, however. We now know that there are many receptors, channels, and enzymes that CBD binds [41,42]. The influences of exposure to CBD are also not limited to suppression of anxiety, and the influences are also not limited to the central nervous system. For example, there are recent studies using CBD as an anti-cancer medicine [43]. Recent studies have also shown that CBD can be a modulator of drug metabolism by binding to the enzymes, CYPs and UGTs. It is necessary to have a broader understanding of the receptors involved, the influences of their activation, and the mechanisms of action. 

When we use phytochemicals, we often face the fact that most of the phytochemicals are hydrophobic (the XlogP3-AA of CBD is 6.5 according to PubChem), which limits their biological availabilities and necessitates administering higher concentrations to obtain the expected effects. CBD can also bind to human serum albumin and form a protein–CBD complex, which will affect its efficacy [44,45,46], which will also necessitate administering a higher dose, but the increase in dose can lead to hepatotoxicity [47]. We also need to consider the chemical stability and the nature of their oxidized products. How to improve the biological availability and how to enhance the stability are important questions because they might lead to decreasing the side effects that some of the phytochemicals have. In the case of CBD, there are some ‘minor to moderate’ side effects reported, such as gastrointestinal symptoms, fatigue, and influences on appetite [48]. Enhancement of biological availability and stability may contribute to suppression of side effects by enabling dosage decreases. This is one of the important tasks to take into consideration when we utilize CBD. 

### 2.2. Brief History of CBD

**Figure 1:**
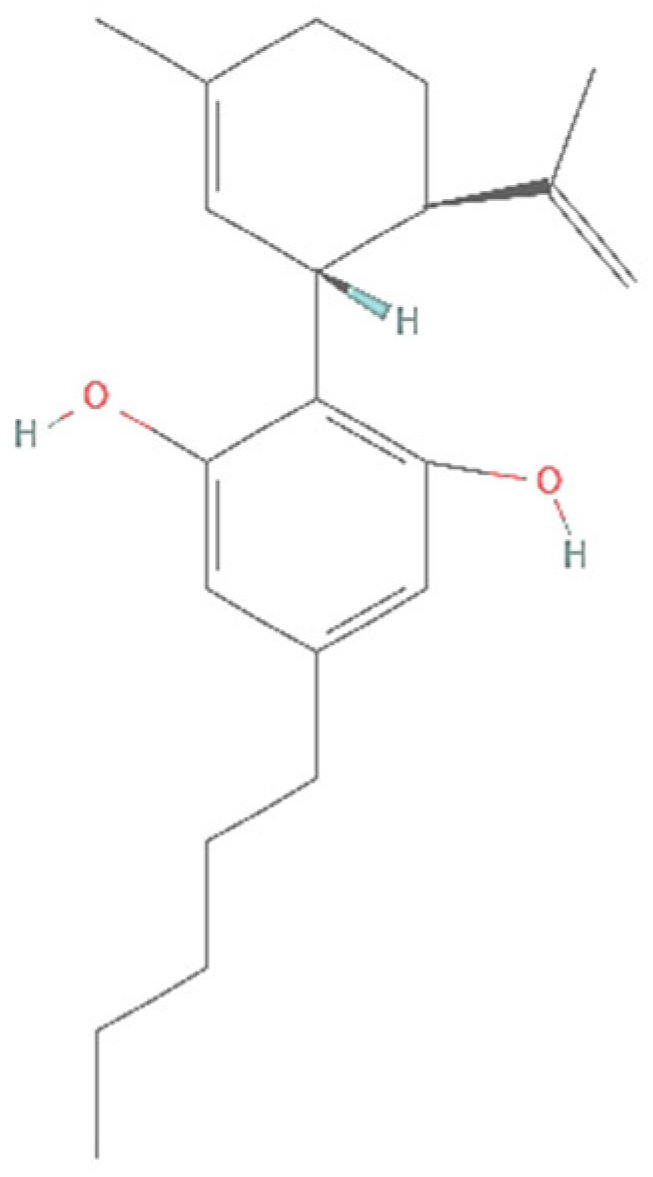
Chemical structure of cannabidiol (CBD) (from PubChem website, PubChem CID: 644019; molecular formular C_21_H_30_O_2_; MW: 314.5 g/mol).

CBD (Figure 1) was first isolated by a group of organic chemists: Adams, Pease, and Clark, in 1940 [49,50], and its structure was determined in 1963 by another group of organic chemists: Mechoulam, Shvo, and Hashish [51]. In 1970, Mechoulam and his collaborators made the important finding that THC is the chemical constituent responsible for the psychoactive responses and that CBD does not have that effect [36,50]. The same group of people, Mechoulam and his collaborators, later identified the endocannabinoids in the 1990s as well [39,40]. Thus, we owe many of the major findings in the history of cannabinoids to Mechoulam and his collaborators [52].

There are many studies on the therapeutic effects of both THC and CBD. This was perhaps because they were considered the two major chemical constituents of cannabis with bioactive properties, although there are many other chemical constituents in cannabis. Historically, cannabis was used to treat convulsions and seizures, which likely influenced early research trends focusing on the effects of THC and CBD on epilepsy, which is a type of disorder in the brain that has seizures as symptoms. There are studies from the 1970s using both THC and CBD, testing and comparing their effects on epilepsy [53,54,55], although the number of studies is not large. A search of PubMed using the keywords ‘CBD and epilepsy’ or ‘CBD and therapeutic’ shows that the number of studies suddenly increased after around 2010, which suggests that the finding of cannabinoid receptors in the beginning of the 21st century contributed to the increase in studies. The stream of studies using CBD alone or CBD and THC since the 1970s accumulated evidence for the positive effects of CBD on epilepsy without major side effects like THC has, and it became officially approved by the Food and Drug Administration (FDA), Silver Spring, MD, USA, in June 2018 to use for the treatment of seizures (“Epidiolex [CBD] oral solution for the treatment of seizures associated with two rare and severe forms of epilepsy, Lennox–Gastaut syndrome and Dravet syndrome, in patients two years of age and older”) [56]. In this history of studies from the 1970s, THC was also found to have anti-convulsant effects (for example, Ham et al. [57]). The finding that THC is the chemical compound involved in producing psychoactive properties with addiction potential has separated the two major chemical constituents of cannabis, i.e., CBD as a chemical compound with a high potential to become a new drug and THC as a chemical compound with problematic side effects.

## 3. Multiple Targets and Multiple Effects of CBD

We tend to think that one type of chemical compound specifically activates a certain type of receptor; however, most biological phenomena are far more complex. We need to think that we are dealing with a complex system, and we are seeing only the surface of it. 

The mechanisms of action of CBD have been unclear, not because the receptors were not found but perhaps because it was found to have binding affinity with various receptors, channels, and enzymes. An in silico study on the binding affinity of CBD listed over 20 possible targets [41]. More than half of these are not experimentally validated yet, and they are predicted to be targets based on the binding affinity. Having a high binding affinity also does not indicate that they will activate the related signaling pathways, and some also inactivate/suppress the signaling pathways. Another study has discussed that there are in total 65 molecular targets that CBD interacts with and that almost half of them are enzymes; others are transporters (20%), ion channels (15%), and receptors were only 15%. Table 1 shows a list of some of the molecular targets that are either known to be activated/inactivated by CBD or have high binding affinity with CBD so that they are hypothesized to be activated/inactivated by CBD. Importantly, although the name cannabidiol may suggest it activates cannabinoid receptors, it is now known to be an antagonist/inverse agonist and allosteric modulator of CB1 and CB2. This means that they bind to outside areas of the regular orthosteric binding site, and this binding to the allosteric binding site could negatively affect other molecules’ binding affinity to CB1 and CB2 receptors and/or suppress the activation of them, even when some other molecules bind at the orthosteric binding site [58]. 

There are multiple effects of CBD reported, i.e., (1) effects that are most likely related to the influences of pathways activated by CBD on the CNS (anti-anxiety, anti-depression, anti-insomnia, anti-epilepsy, anti-convulsant, anti-psychotic, increase in neurogenesis indirectly produced by suppressing anandamide uptake, improvement in cognitive function through increased neurogenesis [59], and others), (2) anti-cancer effects (pro-apoptotic, suppress cell migration and proliferation), (3) anti-bacterial effects (to Gram-positive bacteria such as *Staphylococcus species*, *Listeria monocytogenes*, *Streptococcus mutans*, inhibiting planktonic growth and biofilm formation in a dose-dependent way) [60,61], (4) suppression of inflammation and pain (analgesic), and (5) suppression of drug metabolism. We can see that CBD produces a broad range of its effects by the large number of interacting receptors, channels, and enzymes (Table 1). We can also see that, although the generally known image of the effects of CBD is related to the CNS (group number 1 above), the effects are far beyond that image. Among these, we will especially focus on group number 4 and 5, i.e., the effects of CBD on inflammation, and suppression of drug metabolism in this review to understand how CBD inhibits tacrolimus metabolism and affects the chronic pain that many post-organ-transplant recipients experience.

**Table 1:** Molecular targets of CBD and the effects.

Group	Target	Activate (Agonist)/Inactivate (Antagonist) and Other Actions	Effects	Relevance to Post-Organ-Tranbsplant Care	References
Receptors	Cannabinoid receptor 1 (CB1)	Antagonist [62]; limited affinity [41]; inverse agonist/antagonist and negative allosteric modulator [63]	Anxiolytic effects [64]; increase neurogenesis by indirectly activating CB1 by inhibition of anandamide metabolism/uptake [62]; pain relief [63]; increase neurogenesis [65]. Although CBD is not an agonist of CB1 and CB2, CB1/CB2 antagonist reversed the anti-inflammatory effects of CBD indicating some roles they have on the anti-inflammatory effects by CBD [66].		[41,62,63,64,65,66,67]
	Cannabinoid receptor 2 (CB2)	Inverse agonist [41,63]; antagonist [63]; negative allosteric modulator [63]	Similarly to CB1, CB2 is considered to be involved in the effects of CBD (see above) although CBD is not an agonist of CB2.	Indirect effects of CBD on activation of CBD through suppressing hydrosis of AEA can have analgesic effects that can be effective in post-transplant care.	[41,63,64,67,68]
	G-protein receptor 55 and 18 (GPR55, GPR18)	Antagonist	Anti-epileptic effect [63]; expressed in immune cells other than in the nervous system and tissues/organs. CBD suppresses GPR55 [69]. Activation of GPR55 is known to be pro-inflammatory but it is also found to suppress degranulation of neutrophils and ROS production [70]. (see Section 4.1)		[41,63,67,69,70,71,72]
	Serotonin receptor 1A and 2A (5-HT_1A_ and 5-HT_2A_)	Agonist	Anxiolytic effect [62,63,64], vertical motor effect at high dose [73]; anti-epileptic, antipsychotic effect [63]; antidepressive effect [63]; improve sleep [74]	Anxiolytic effects and anti-depressive effect may have positive effects after organ-transplant	[41,62,63,64,67,71,73,74]
	Adenosine receptor (AR)	Agonist	Anti-inflammatory effects through activation of A_1_ and A_2_AR, and inhibition of A_2_AR results in reduction in pro-inflammatory cytokines such as TNFα [71]. Performed via suppression of equilibrative nucleoside transporter (ENT) resulting in enhanced adenosine signaling. The anti-arrhythmic effects seen with A_1_ receptor activation are mediated through the same pathway [67]. Suppresses the function of nucleoside transporter which uptakes adenosine, and enhances adenosine signaling, resulting in producing anti-inflammatory and immunosuppressive effects [66]	Anti-inflammatory effects may improve conditions after organ-transplant	[66,67,71,75]
	Peroxisome proliferator-activated receptor gamma (PPARγ)	Agonist	Anxiolytic effect, anti-inflammatory effects; CBD binds to the ligand binding domain of PPARγ and PPARγ translocate with RXR. PPARγ/RXR binds with a specific DNA region called the peroxisome proliferator hormone response elements (PPRE) and produces transrepression effects, suppressing the expression of pro-inflammatory genes (see Section 4.1)	Anti-inflammatory effects may improve conditions after organ-transplant	[63,64,67,71,76]
	Dopamine receptor (D)	Partial agonist of D2	Functioned similarly to aripiprazole, an antipsychotic drug, inhibiting the binding of dopamine antagonist domperidone [77]		[41,63,77]
	Glycine receptor alpha1, alpha 3 (GlyRα1, α3)	Agonist	Activated GlyR α3 5 times stronger than glycine. Effects in detail need to be addressed.		[67]
Channels	Transient receptor potential cation channel vanilloid subtype 1 (TRPV1)	Agonist; also indirectly activates by increasing the level of TRPV1 agonist AEA [78]	Antipsychotic effect [62,63], anti-inflammatory, anti-hyperalgesia effects [79,80]; CBD increases myeloid-derived suppressor cells (MDSC) which produce arginase and suppress NK cells, B cells, and T cells [81]	Anti-inflammatory effects may improve conditions after organ-transplant	[41,63,71,75,78,79,80,81,82,83,84,85]
	Transient receptor potential cation channel vanilloid subtype 2 (TRPV2)	Agonist; CBD is most effective activating TRPV2 among cannabinoids [84]	Anti-cancer [80,86]; becomes desensitized after activation similarly to TRPV1 [80]	Similar to TRPV1	[41,63,68,71,75,80,83,84,86,87,88]
	Transient receptor potential cation channel vanilloid subtype 3 and 4 (TRPV3 and TRPV4)	Agonist	TRPV3: Mostly expressed in the brain and in skin and tongue, efficacy to activate is less than TRPV1 and TRPV2 [68]; TRPV4: least effective in activating compared to other TRPs, anti-acne effect possibility, induce glioma cell death [80]		[63,68,80,89]
	Transient receptor potential cation channel ankyrin subtype A1 (TRPA1)	Agonist	Excitatory effects on vagal afferent neurons [90]		[71,83,90,91]
	Transient receptor potential cation channel melastatin type 8 (TRPM8)	Antagonist	Possibly involved in anti-cancer effects of CBD		[63,68,71,84,91]
	T-type Ca^2+^ channel	Antagonist	Anti-epileptic effects by blocking low-voltage T-type Ca^2+^ channels, which is involved in absence epilepsy (short periods of blanking out or lapse of consciousness, lasting less than 20 s)		[63,71]
	Kv7 channel	Agonist	Activate Kv7 at 30 nM		[92]
Proteins/enzymes	Cytochrome P450 (CYP)	Enzymes/proteins Antagonist	Suppress drug metabolism by binding to CYP enzymes mainly CYP2C9, CYP2C19, and CYP3A4 or CYP3A5 (see Section 5)	CBD inhibits CYP and suppress drug metabolism	[41,93,94,95,96]
	Uridine 5′-diphospho-glucuronosyltransferase (UGT)	Antagonist	Suppress drug metabolism by binding to UGT (see Section 5)	CBD inhibits UGT and suppress drug metabolism	[12]
	Fatty acid amide hydrolase (FAAH)	Antagonist	Antipsychotic effect [63]; improve sleep [63]; Binding of CBD to FAAH suppresses the hydrolysis of AEA by FAAH, which suppresses lead to suppression of prostaglandin E2 (PGE2), a key inflammatory mediator. This can lead to the analgesic effects by CBD [97]	CBD binding to FAAH can suppress AEA hydrolysis, which can lead to activation of CB1/CB2	[63,67,97]
	Fatty acid binding protein (FABP)	Agonist	Intracellular transporter of CBD. Suppress endocannabinoid metabolism [98]. (see Section 4.1 on CB1/CB2)	CBD binding to FABP can suppress AEA hydrolysis, which can lead to activation of CB1/CB2	[41,98]
	Interferon (IFN)γ	Antagonist [63]	Attenuated expression of interferon stimulated genes [63]; Suppressed IFN γ production and IL2 in splenic T cells [99];	Anti-inflammatory effects may improve conditions after organ-transplant	[41,63,75,99,100]
	γ Aminobutyric acid (GABA_A_)	Agonist	Anti-epileptic effect		[63,71]

## 4. Anti-Inflammatory Effects of CBD

As shown in Table 1, CBD interacts with a wide range of receptors, channels, and enzymes. The anti-inflammatory effects of CBD can target immune cells and non-immune cells and interfere with specific molecules involved in the inflammatory signaling pathways. Notably, multiple pathways often work synergistically to produce CBD’s anti-inflammatory outcomes. Here, we will focus on the several routes (or pathways) where CBD exerts its anti-inflammatory effects, specifically those related to organ systems outside the CNS.

### 4.1. Representative Molecular Targets and Signaling Pathways

First we will summarize some of the receptors and channels with which CBD is known to interact. 

#### 4.1.1. TRPV1

**Figure 2:**
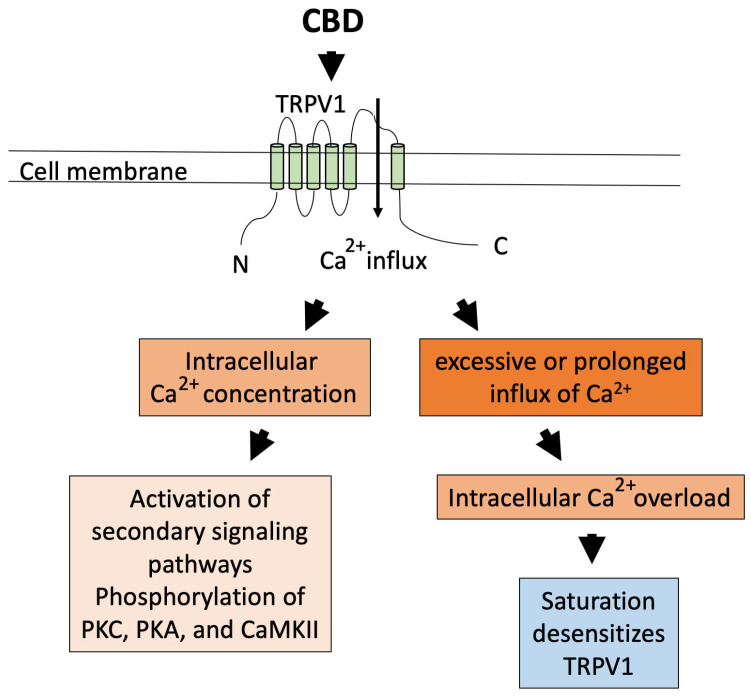
Hypothetical mechanisms of action of anti-inflammatory effects by exposure to CBD through TRPV1. Activation of TRPV1 and desensitizing TRPV1 in neuronal and non-neuronal cells. Activation of the TRPV1 channel triggers Ca^2+^ influx, which leads to increased intracellular calcium concentration and initiation of secondary signaling pathways, stimulating PKC, PKA, and CaMKII. Overload of Ca^2+^ or prolonged influx of Ca^2+^, dephosphorylation of the channel takes place, and the channel becomes unable to respond to further stimulation, desensitizing the TRPV1 channel [80,103,104,105,106,107,108,109]. PKC: protein kinase C, PKA: protein kinase A, CaMKII: calmodulin-dependent protein kinase II.

Transient receptor potential vanilloid (TRPV) 1 is a member of the transient receptor potential (TRP) channel superfamily. The effects of CBD may be mediated through TRPV1 and one of the serotonin receptors, 5-HT1A [97]. 5-HT1A is expressed mainly in the CNS, whereas the TRPV1 channel is well known to be expressed in both pain sensory neurons to detect heat, acidity, mechanical stress, and spiciness but also expressed in various types of immune and non-immune cells [66,81]. Activation of the TRPV1 channel produces an influx of monovalent and divalent cations and specifically a Ca^2+^ influx (Figure 2). In a study using Caco-2 cells established from colorectal adenocarcinoma cells and human colonic explants, CBD suppressed the inflammatory responses of cells pre-stimulated with either IFNγ or TNFα [85]. When the TRPV1 antagonist SB366791 was added with CBD, the anti-inflammatory effects of CBD were blocked, indicating the role of TRPV1 in the anti-inflammatory effects of CBD [85]. In a mouse model of experimentally induced hepatitis (C57BL/6 mice), CBD dose-dependently reduced inflammatory biomarkers and suppressed hepatitis. However, these effects were absent in TRPV1 knockout mice, further supporting the involvement of TRPV1 [81]. Importantly, their study showed that these anti-inflammatory effects of CBD in the mice with hepatitis were mediated by the CBD-induced increase in the arginase-expressing CD11b^+^Gr-1^+^ myeloid-derived suppressor cells (MDSCs) [81]. CD11b is a marker for myeloid cells of the monocyte/macrophage lineage. Gr-1, or granulocyte receptor-1 antigen, is a marker for granulocytes, and MDSCs are known to suppress natural killer (NK) cells, B cells, and T cells by producing arginase. Arginase down-regulates the expression of the TCR component CD3 [101], and down-regulation of CD3 impairs signaling, thus increasing the CD11b^+^Gr-1^+^ MDSC cell population, which becomes immunosuppressive [102]. This indicates that one of the anti-inflammatory effects of CBD on a pathway can be indirect through the activation of TRPV1 and increasing the CD11b^+^Gr-1^+^ MDSC. 

The TRPV1 receptor is known to have a gustatory role in detecting spice in foods. Capsaicin, present in chili peppers, is one of the phytochemical compounds that is well-known as a ligand of TRPV1 [84,110], and the sense of spiciness of chili pepper is mediated by capsaicin activation of TRPV1 [111]. If the anti-inflammatory effects of CBD are mediated by activation of TRPV1, it is possible that capsaicin may also have anti-inflammatory effects. In fact, there are studies showing the anti-inflammatory effects of capsaicin (capsaicin inhibits the enzymatic activity of CYP dose-dependently as well) [20,112,113]. 

As TRPV1 is a receptor channel involved in sensing spiciness, heat, and irritation, which are overall noxious sensations, a key question remains as to why activation of TRPV1 is anti-inflammatory. Indeed, there are conflicting studies reporting that activation of TRPV1 has pro-inflammatory effects and causes pain [114,115,116], while other studies report its anti-inflammatory effects [79,117] and also studies reporting both [115]. One of the hypotheses to explain the anti-inflammatory effects is ‘paradoxical analgesic activity’, which hypothesizes that, when administered at high(er) concentrations, the binding of CBD to TRPV1 leads to its activation, which triggers Ca^2+^ influx, initiating multiple secondary signaling pathways, stimulating phosphorylation of protein kinase C, protein kinase A, and Ca^2+^/calmodulin-dependent protein kinase II, and when there is excessive or prolonged influx of Ca^2+^, it leads to dephosphorylation of the channel, and the channel becomes unable to respond to further stimulation (desensitization of the channel) [68,78,84,104,118,119,120] (Figure 2). There is also a hypothesis, by a study on TRPV2, that CBD binds to a specific site of the hydrophobic pocket of the channel, and next to this site, the gate for Ca^2+^ influx opens, suggesting that depending on the site of binding, the signaling becomes modified [118]. Confirming these hypotheses requires future investigation.

Thus, although there are still details that need more study, it is plausible that CBD mediates its anti-inflammatory effects through TRPV1 activation. 

#### 4.1.2. PPARγ

**Figure 3:**
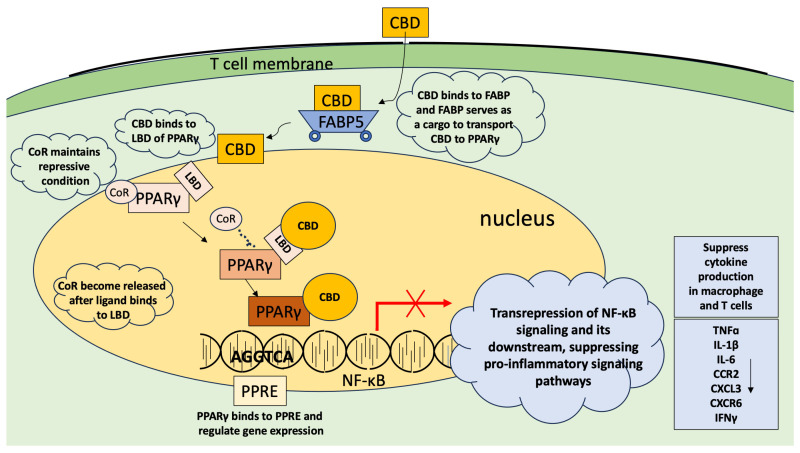
Anti-inflammatory effect of CBD through activation of PPARγ. FABP (especially FABP5) serves as an intracellular transporter of CBD. CBD binds to the ligand-binding domain (LBD) of PPARγ, which initiates transrepression with a specific DNA region called the peroxisome proliferator hormone response element (PPRE) and produces transrepression effects, suppressing the transcription activities of NF-κB and the nuclear factor of activated T cells (NFAT), etc., and blocks the downstream signaling of them, suppressing the expression of, for example, IL-2, TNFα, IL-1β, IL-6, MMP9, and IFNγ [121,134,135,136]. Arrow in the grey box show the decrease in the amount. Other arrows show the process.

Peroxisome proliferator-activated receptor (PPAR) proteins are members of a nuclear hormone receptor superfamily, expressed in various metabolically active tissues such as adipose tissue, liver, and heart. They are also expressed in macrophages, dendritic cells, B cells, and T cells [121,122,123,124]. There are three types of PPARs, i.e., PPARα, PPARβ/δ, and PPARγ. Activation of PPARβ/δ enhances metabolism of fatty acids and maintains lipid homeostasis [125,126] and all three types of PPARs play significant roles in controlling inflammation [121,127]. Although PPARα and PPARγ are both known to be expressed in lymphocytes [123,124], there is evidence showing that PPARγ is involved in the anti-inflammatory effects of CBD [128]. It is well-established that activation of PPAR produces anti-inflammatory effects [129,130] and the mechanism of action is known in detail. There are multiple mechanisms of known actions. One is a transactivation mechanism in immune cells following ligand binding. In this scenario, PPAR makes an obligate heterodimer with retinoid X receptors (RXR), and, without the ligand binding, there is a co-repressor bound to the PPAR/RXR complex, tran-srepressing genes [130,131,132]. Ligand binding stimulates conformational changes, releasing the corepressor bound to the heterodimer complex, and initiates transactivation. In another mechanism of action, which is considered more related to immune cells, PPARγ functions as a monomer and transrepresses pro-inflammatory genes. The activation starts from the binding of the ligand (for example, CBD) to the ligand-binding domain of PPAR (LBD in Figure 3), which comprises 13 α helices and a four-stranded β sheet, forming a rather large pocket structure as a ligand-binding domain [129]. This large binding domain enhances the flexibility of PPAR, enabling various chemicals to become its ligands. Activation of PPAR receptors produces anti-inflammatory effects by inhibiting other transcription factors, such as nuclear factor kappa light-chain-enhancer of activated B cells (NF-κB), activator protein-1 (AP-1), and signal transducer and activator of transcription (STAT) [121,129,130,133]. Activated PPAR seems to directly interact with NF-κB between the ligand-binding domain of PPAR and the p65 subunit of NF-κB, and this not only inhibits NF-κB signaling but also induces degradation of the NF-κB/p65, thus inhibiting the expression of pro-inflammatory cytokines and chemockines/chemokine receptors such as COX2, TNFα, IL-1β, IL-6, and others [78] (Figure 3).

Various phytochemicals are ligands of PPARs. Focusing on PPARγ alone, which is involved in the anti-inflammatory effects of CBD, there are, for example, β-caryophyllene, carnosic acid, carnosol, curcumin, piperine, resveratrol, kaempferol, naringenin, genistein, and many others [137,138,139,140,141,142,143,144]. This large range of phytochemicals that activate PPARγ illustrates the diversity of phytochemicals with anti-inflammatory effects. 

PPARs are nuclear receptors and not membrane receptors. A recent study revealed that fatty acid binding protein (FABP) serves as an intracellular transporter of CBD to the nucleus where PPARγ receptors are located [98]. There are multiple types of FABP, and macrophages and dendritic cells express FABP4 and 5 mainly, whereas T cells express FABP5 predominantly [145]. The level of expression also changes depending on the conditions. Naïve CD4^+^ and CD8^+^ T cells express FABP5 in the main, and the level was found to be highest in CD8^+^ T cells in an exhausted state [145], which is a dysfunctional state of T cells caused by, for example, chronic infection [146]. As the name suggests, it serves as a lipid chaperone, binding to hydrophobic ligands such as fatty acids, hence the name [147]. Elmes et al. [98] have shown that CBD has high binding affinity with FABP3, FABP5, and FABP7, although it is not as high as the fatty acid, oleic acid, which showed 50 times higher binding affinity with FABPs than CBD showed [98].

#### 4.1.3. GPR55

**Figure 4:**
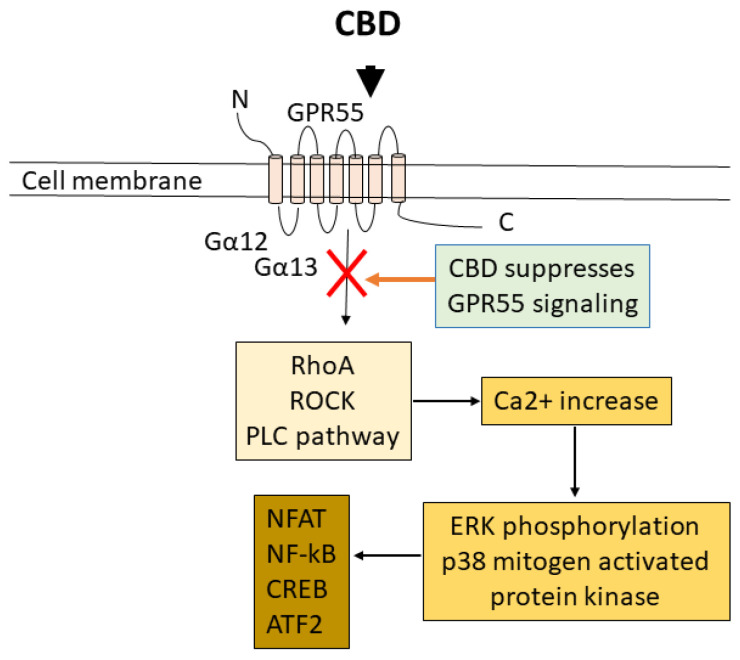
GPR55 is a seven-transmembrane receptor coupled to Gα12,13 proteins. It activates RhoA and ROCK and the PLC pathway, which causes an increase in intracellular Ca^2+^. Increase of Ca^2+^ triggers phosphorylation of ERK, activates p38 mitogen-activated protein kinase, and the downstream signaling of NFAT, NF-κB, CREB, ATF2.

Similarly to CB1 and CB2, GPR55 is a member of the seven-transmembrane G protein-coupled receptor family that senses lipids and peptides [148]. It shares many cannabinoid ligands with CB1 and CB2, and thus it is also called the 3rd cannabinoid receptor (CB3) [149,150]. It is involved in various physiological functions such as regulation of dorsal root ganglia excitability and inflammatory and neuropathic pain, neutrophil migration, bone metabolism, angiogenesis, renal tubule hypertrophy, and so on [151]. It is expressed in the nervous system as well as other tissues and organs, and it is expressed in the immune system [151,152]. In the immune system, it is expressed in B and T cells, and dendritic cells in mouse spleen [152], and, in humans, it is expressed in B and T cells, macrophages, and neutrophils [151,153]. In a study that focused on peripheral blood cells, GPR55 was predominantly expressed in the monocytes and NK cells, but was also expressed in myeloid dendritic cells, B cells, Treg cells, and T cells, in this order [154]. Importantly, the expression of GPR55 becomes upregulated when immune cells are activated [148], and is considered to have a pro-inflammatory function. It was found upregulated in resting B cells upon viral or bacterial infection and in CD4^+^ and CD8^+^ T cells upon exposure to CD3 and CD28 [148]. CBD is known to suppress GPR55 [69]. GPR55 is coupled to Gα12,13 proteins and activates the ras homolog gene family member A (RhoA) and Rho-associated protein kinase (ROCK), which elicits the phospholipase C (PLC) pathway to increase intracellular Ca^2+^, which triggers extracellular regulated kinase (ERK) phosphorylation and its downstream pathway (Figure 4). 

#### 4.1.4. CB1/CB2

We briefly wrote about cannabinoid receptors 1 and 2 (CB1 and CB2) at the beginning of this review. Here, we will focus on their expression in the immune system and the signaling pathway. CB1 is mostly expressed in the CNS, but it is also expressed less abundantly in various tissues [155]. It is also found, although much less, in human leukocytes [156]. The expression among leukocytes was highest in B cells, followed by eosinophils, granulocytes, and very few in T cells and monocytes [156]. For many years, CB2 has been known as the cannabinoid receptor expressed in peripheral nerves and in the immune system. This was discovered soon after the cannabinoid receptors were found. In a recent study comparing expression among the types of immune cells, it was found in high amounts in the eosinophils and then B cells, but in very low amounts in other types of immune cells [156]. Turcotte et al. [157] conducted a literature search on the expression of CB2 in different types of immune cells. The types of immune cells that were reported in multiple papers to express CB2 were B cells, monocytes, neutrophils, and T cells, and, interestingly, multiple papers reported that neutrophils do not express CB2 [157]; thus neutrophils were reported positively and negatively in multiple papers. It has been well-documented in the neuronal cells that the expression of CB2 is controversial [158], and one reason was because of the lack of reliable antibodies [159] and another reason is possibly because CB2 is expressed only when there is inflammation [160]. It could be that the same is happening in the case of CB2 expression in neutrophils. This needs to be determined in future studies. 

**Figure 5:**
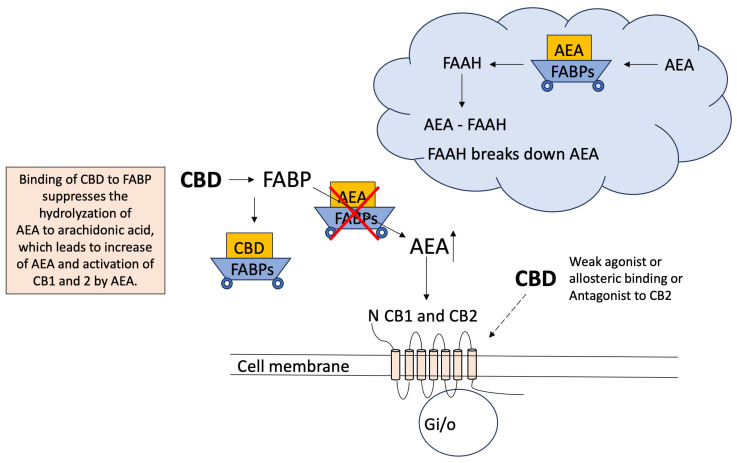
Indirect influences of increasing the activation of CB1 and CB2 through competitive binding to FABP. CBD is an antagonist/inverse agonist and allosteric modulator of CB1 and CB2, and this can negatively affect other molecules’ binding affinity to CB1 and CB2 receptors [58]. However, CBD can indirectly activate CB1 and CB2 by binding to FABP, suppressing the binding of FABP to AEA (illustrated on top right), and increasing the levels of AEA, which binds and activates CB1 and CB2. FABP: fatty acid binding protein, AEA: anandamide, FAAH: fatty acid amide hydrolase. The arrow next to AEA show the increase in the amount and other arrows show the process.

There have been many studies on the anti-inflammatory effects of CB2. Activation of CB2 is known to have not only anti-inflammatory effects but also analgesic, anti-nociceptive, antioxidant, anti-tumor, anti-ischemic, and neuroprotective effects (there are many references, for example, a recent review [161]). CBD is not an agonist for CB1 or CB2 (see Table 1). It is known to have weak, limited binding affinity and is also an antagonist or inverse agonist or negative allosteric modulator of both CB1 and CB2. Also, how it interacts with these receptors seems to depend on its concentration (Table 1). There are also some studies showing that CB1 antagonist and CB2 antagonist reversed the anti-inflammatory effects of CBD [66], indicating that, even if they are not directly involved, CB1 and CB2 seem to be somehow involved in CBD’s anti-inflammatory effects. There are studies indicating that CBD indirectly activates CB1 and CB2 by increasing the levels of one of the endocannabinoids, AEA, which binds and activates CB1 and CB2 by binding to FABP. FABP serves as a carrier of AEA (as well, other than CBD) and transports it to fatty acid amide hydrolase (FAAH), which breaks down AEA by hydrolyzing AEA to arachidonic acid [162]; thus, competitive binding of CBD to FABP can suppress the breakdown of AEA and increase the levels of AEA, leading to an increase in the activation of CB1/CB2 and producing anti-inflammatory effects (Figure 5). 

#### 4.1.5. Adenosine Receptor

**Figure 6:**
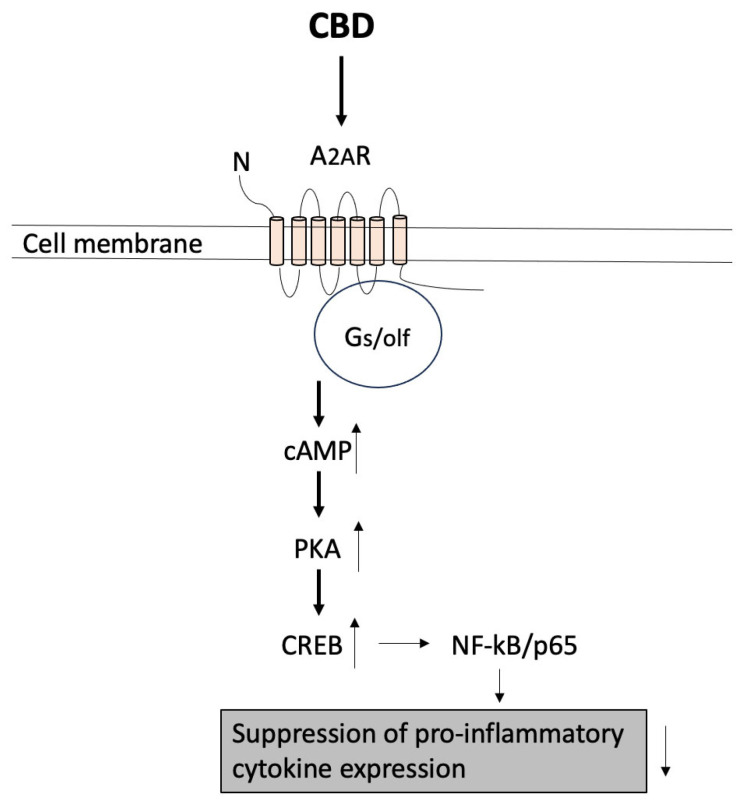
Effects of CBD on adenosine receptors. Binding of CBD to the adenosine receptors increases the intracellular cAMP levels, the levels of PKA, and activation of CREB [163]. This will lead to the suppression of transcriptional activity of NF-κB/p65 and suppression of pro-inflammatory cytokine expression [163]. Thick arrows indicate the process, and thin arrows show increase/decrease in the amount.

The adenosine receptor is a transmembrane G protein-coupled receptor. There are several sub-types, which are A_1_, A_2A_, A_2B_, and A_3_. These groupings are based on the way they are coupled with G-proteins. A_1_ and A_3_ are coupled with G_i/o_, whereas A_2A_ and A_2B_ are coupled with G_s/olf_. These four subtypes have differences in their characteristics; for example, A_1_ and A_2A_ become activated by low levels of adenosine (0.01 to 1 μM), whereas A_2B_ and A_3_ become activated by rather higher concentrations of adenosine (over 10 μM) [163]. Adenosine receptors are all expressed in immune cells and involved in regulating inflammation, although their expression depends on the types of cells as well as the ‘environmental conditions’; for example, IL-1 and TNFα increase the A_2A_ receptor expression in monocytes [164]. CBD is known to activate A_2A_ [66,72,75] and activation of A_2A_ by CBD is known to have anti-inflammatory effects [66,67,71,75]. The signaling pathways that become activated by activation of A_2A_ are well established. It leads to an increase in the intracellular cAMP levels, which stimulates cAMP-dependent protein kinase (PKA), which activates nuclear substrate cAMP-responsive element-binding protein (CREB) [163]. CREB regulates gene expression in a way that transcriptional activity of NF-κB/p65 becomes suppressed and leads to suppression of pro-inflammatory cytokine expression [163] (Figure 6). 

Interestingly, there is a lack of recent studies investigating the binding affinity or activation of adenosine receptors by phytochemicals. A search of the literature identified only three studies, all conducted by the same group between 1996 and 2002, on the interactions of phytochemicals with adenosine receptors [165,166,167]. These studies reported that, for example, galangin, which is a chemical constituent of propolis, bound to A_1_, A_2A_, and A_3_ receptors at μM levels, and genistein, which is found in soybeans, has binding affinity with A_1_ receptor [165]. 

#### 4.1.6. Inflammatory Signaling Pathways Affected by CBD

**Figure 7:**
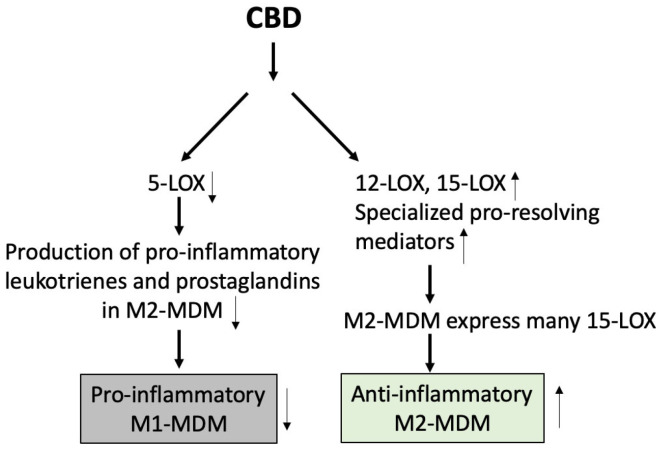
Influence of CBD on molecular switch to produce anti-inflammatory M2-monocyte-derived-macrophages. CBD increases specialized pro-resolving mediators by stimulating phospholipase A2 enzyme-dependent polyunsaturated fatty acid release and by increasing 12/15-LOX. It also suppresses 5-LOX leukotriene production [16]. M2-MDM: M2-monocyte-derived macrophage, LOX: lipoxygenase. Thick arrows show the process, and thin arrows show increase/decrease in the amount.

A recent study has also shown that CBD could function as a lipid mediator class-switching agent that suppresses eicosanoid biosynthesis, which produces pro-inflammatory leukotrienes and prostaglandins, and increases pro-resolving mediators such as resolvins and protectins in the immune cells through stimulation of phospholipase A2-dependent polyunsaturated fatty acid release [16]. Peltner et al. [16] suggested that these changes could increase anti-inflammatory M2 monocyte-derived macrophages rather than the pro-inflammatory M1 monocyte-derived macrophages (Figure 7) and suggested that this could be the mechanism of action for the anti-inflammatory effects of CBD.

In Figure 2, Figure 3, Figure 4, Figure 5, Figure 6 and Figure 7, we illustrated the receptors, channels, and possible switch in the pathways that can be activated (TRPV1 and PPARγ) or inactivated/suppressed (GPR55) by exposure to CBD, directly (TRPV1 and PPARγ) or indirectly (CB1/CB2). CBD’s multiple targets for interaction and the various effects produced from interacting with these targets reveal the complexity of its immunosuppressive effect. It is very possible that its effects are produced synergistically through activating or inactivating multiple targets. 

There are many studies showing the influences of CBD on signaling pathways and its effects on inflammatory cytokines, of which some are summarized in Table 2. 

## 5. Binding of Cannabidiol to CYP and UGT Enzymes

CYP enzymes play a central role in Phase I metabolism by facilitating the oxidation and hydroxylation of xenobiotics, whereas UGT enzymes are involved in glucuronidation in Phase II metabolism. CYP and UGT together metabolize over 90% of the drugs and xenobiotics, such as chemical compounds from the environment and food, and CYP has the larger role in metabolism as it is involved in the first phase of metabolism [7,171,172]. CBD was found to suppress the metabolism of many drugs by binding with CYP and UGT [173], i.e., becoming the substrate to be metabolized and suppressing the metabolism of drugs that were co-administered. There are several mechanisms that can produce this: the chemicals with higher binding affinity can occupy CYP’s active binding site (competitive binding) and go through the metabolizing process, and thus the other drug remains unmetabolized. Another way is that one type of chemical binds at an allosteric binding site and modifies the shape of CYP. This allosteric binding will either allow another chemical to bind at the orthostatic binding site but be metabolized less, due to the allosteric modifications, or the other chemical is unable to bind at the orthostatic binding site due to the modified shape [174].

### 5.1. The Early Findings on the Inhibition of CYP by CBD

From as early as the 1970s, soon after the identification of THC and CBD, there have been studies on the effects of CBD on co-administered drugs. For example, Paton and Pertwee [175] reported that pentobarbitone (the same as pentobarbital)-induced sleep/immobilization in mice was extended when cannabis extract, CBD, or THC was co-administered [175]. The effect of CBD on extending sleep was stronger dose-dependently and with 50 mg/kgbw of CBD administration, the time length of pentobarbitone-induced sleep/immobilization was more than three times longer than the control group of mice that received only pentobarbitone. In their study they also compared the effects of cannabis extracts, CBD, and THC on inhibiting the metabolism of phenazone (analgesic and anti-inflammatory drug). Supernatant of mouse liver homogenate was exposed to cannabis extract, CBD, and THC in order to determine whether the extended function of pentobarbitone was due to a suppression of pentobarbitone metabolism, and they found a dose-of-CBD-dependent inhibition of phenazone metabolism [175]. Later, Watanabe et al. [176] showed that CBD and THC both significantly suppressed lipid peroxidation in hepatic microsomes and reduced the amount of CYP in microsomes [176]. 

Interestingly and importantly, there were differences in the CYP enzymes that CBD and THC inhibited: CBD inhibited CYP1A1, CYP2B6, CYP2C8, CY2C9, CYP2C19, CYP2D6, CYP2E1, and CYP3A4, CYP3A5, in main [94,177,178], whereas THC inhibited CYP1A2, CYP2B6, CYP2C9, and CYP2D6 [94,95]. Among the CYP enzymes, CYP2C19 and CYP3A4 are considered to be the major CYP enzymes that CBD suppresses [94,95] whereas CYP2C9 and CYP2C19 are the major CYP enzymes for Phase I metabolism that THC suppresses [95]. Such specificities suggested that, if there are genetic differences in the type of CYPs that each person possesses or possesses relatively more, there might be differences in the rate that CBD inhibits CYP. Zhou et al. [179] reported significant ethnic variability in CYP2D6 expression, a CYP subtype inhibited by both CBD and THC [179]. Approximately 5 to 14% of the Caucasian population lacked CYP2D6, whereas almost no African and Asian populations exhibited this deficiency [179]. These genetic differences could affect the effects of CBD on inhibiting CYP and indicate the importance of determining the genotype of patients in clinical situations in administering CBD as well as drugs (see Section 5.6).

### 5.2. Metabolism of CBD by CYP

**Figure 8:**
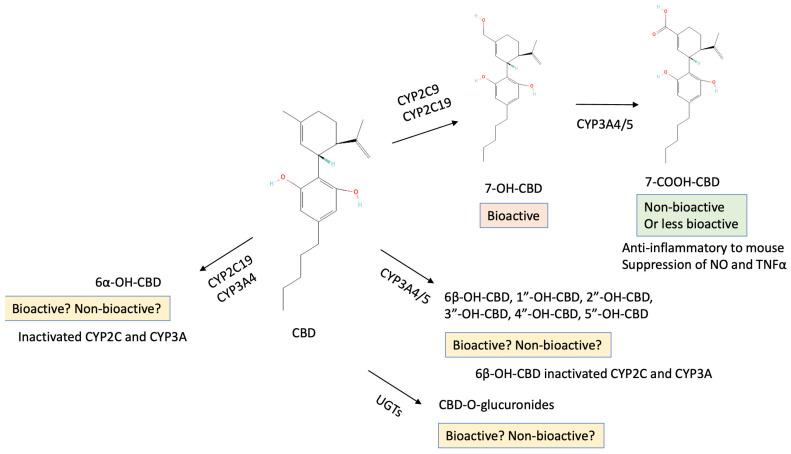
Depending on the types of CYPs that metabolize CBD, the derivatives are different. Bioactive properties of them are not thoroughly determined yet, and some show contradictory results.

During the CYP-mediated metabolism, CBD becomes hydroxylated by mainly CYP2C9, CYP2C19, and CYP3A4 to two major metabolites, i.e., 7-hydroxy-CBD (7-OH-CBD) and 7-carboxy-CBD (7-COOH-CBD) [94,95,96] (Figure 8). Importantly, 7-OH-CBD possesses bioactive properties to inhibit CYP enzymes as similarly as CBD does, although the activity inhibition is CYP-dependent [95,180]. 7-COOH-CBD is reported to be non-bioactive, but there are reports on the anti-inflammatory effects of 7-COOH-CBD in mice [181]. It is important to note that depending on the CYP subtype, CBD becomes metabolized into different chemical compounds. Beers et al. [96] reported that CYP2C9 and CYP2C19 metabolize CBD and form bioactive 7-OH-CBD, whereas binding of CBD with CYP3A4 leads to the formation of 6α-OH-CBD as well as several other metabolites (such as 6β-OH-CBD, 1″-, 2″-, 3″-, 4″-, 5″-OH-CBD). An earlier report by Jiang et al. [177] also showed that CYP2C19 catalyzes CBD to 7-OH-CBD, whereas CYP3A4 metabolizes CBD to 6α-OH-CBD and others. The bioactive properties of these CBD derivatives are not thoroughly understood yet, and this would be especially important when the metabolized compound is bioactive as well as CBD, because if it is bioactive, the effects of administering CBD can be considered ongoing (Figure 8).

### 5.3. Metabolism of Tacrolimus by CYP

**Figure 9:**
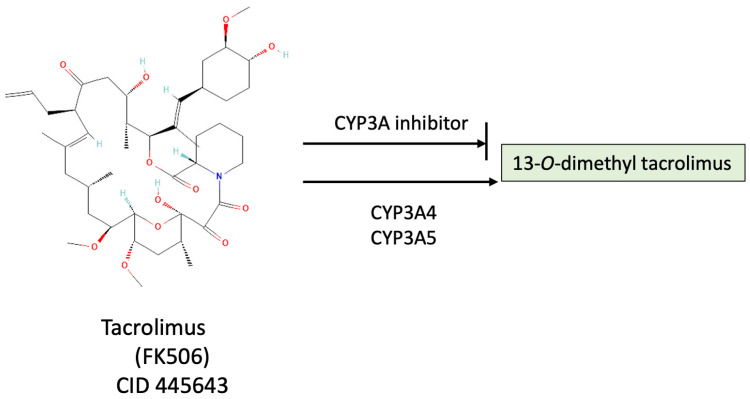
Chemical structure of tacrolimus (FK506) (from PubChem website, PubChem CID: 445643; molecular formula C_44_H_69_NO_12_; MW: 804.0 g/mol). Inhibitors of CYP3A, such as ketoconazole, cyclosporin A, nifedipine [183], SKF525A, troleandomycin suppressed metabolism of tacrolimus (FK506) [185].

Tacrolimus (FK506; CID 445643) (Figure 9) was first found and isolated from a soil bacteria, *Streptomyces tsukubaensis* [182,183,184]. It is one of the drugs used as an immunosuppressant. It becomes metabolized by liver microsomes, and studies comparing CYP subtypes have found that the CYP3A subtype of CYPs (CYP3A4 and CYP3A5) is the major subtype of CYPs involved in the metabolism of tacrolimus [183,185] (Figure 9). In an early study comparing differences in the metabolism of tacrolimus, large individual differences in the formation of tacrolimus metabolite 13-O-demethyl tacrolimus were reported (24.1 to 110.4 pM of 13-O-demethyl tacrolimus/min/mg microsomal protein), and sex differences (F > M) were also found [182]. These substantial interindividual differences have prompted investigations into the genetic variations among patients prescribed tacrolimus. Understanding a patient’s CYP genotype, particularly variations in CYP3A4 and CYP3A5 [186,187], is critical for optimizing tacrolimus dosing to ensure therapeutic efficacy while minimizing toxicity. 

### 5.4. Phase II Metabolism by UGT

While studies on CBD’s effects on UGT-mediated metabolism are fewer compared to its interactions with CYP enzymes, existing research indicates that CBD inhibits glucuronidation activity mediated by UGT [11,12]. Similarly to CYP, there are subfamilies in UGT, and UGT1, UGT2, UGT3, and UGT8 are most involved in drug metabolism [174]. Of these subfamilies, mostly the UGT1 family is involved in the glucuronidation of CBD (UGT1A3, 1A7, 1A8, 1A9 and 2B7) [188]. There are again some discrepancies in the reports on the interaction of CBD with UGT, although UGT1A9 and 2B7 can be considered common to each study [173,188,189,190]. 

### 5.5. Clinical Trials Co-Administrating CBD with Drugs

Multiple studies have evaluated the effects of co-administering CBD with some other drugs (Table 3). Leino et al. [191] reported a case study that co-administering CBD with tacrolimus increased the concentration of tacrolimus in the blood by three-fold. The dose of tacrolimus was stopped or reduced along a time course until it became reduced to 10% of the dose before the prescription of CBD started in order to avoid tacrolimus becoming toxic because of its increase in the system [191]. In their study, the participant took a total amount of 2000 mg of CBD per day (20 mg/kg body weight/day), splitting the total dose in half and taking it twice per day. The participant was a patient with refractory epilepsy, and they reported that the seizure events decreased after the prescription of CBD [191]. In a clinical trial with healthy participants [192], participants daily received 1500 mg of CBD for up to 3.5 weeks, and an elevation of serum alanine aminotransferase (ALT) was observed exceeding the upper limit of normal (ULN) in 44% of the participants, and 33% reached the level of drug-induced liver injury. This suggests that, with post-transplant patients who are immunocompromised or receiving hepatotoxic drugs such as tacrolimus, there is a higher possibility that CBD may cause liver injury. A careful monitoring of the liver function will be necessary, and strategies to reduce the dose of CBD necessary to use to expect the beneficial effects of CBD will be important (see Section 6.2). 

Other studies in Table 3 are either clinical trial phase I with healthy adult participants or reviews. In general, the clinical trial studies mostly reported the effects of CBD on increasing the concentration of drugs in the system (N-desmethylclobazam [193], clobazam and stiripentol [194], and caffeine and paraxanthine [195]).

### 5.6. Polymorphism of CYP and Its Influences on Drug Metabolism

As summarized above, one of the important recent findings is that there are genetic differences in the types of CYP enzymes each person possesses [201]. These differences influence the specific CYP enzyme responsible for metabolizing drugs, including tacrolimus and CBD. Such variability underscores the complex interplay in drug metabolism, driven by individual genetic makeup. There are also sex differences (higher expression of CYP in females than males) and age (greater CYP activity in younger individuals than older individuals) further contribute to variability in CYP enzyme expression and its impact on drug metabolism [202]. 

Recent studies have found an additional factor that affects metabolism by CYP, i.e., the polymorphism of CYP. Solus et al. [201] conducted a comprehensive analysis of genes and reported that they identified 388 different polymorphisms in 11 isoenzyme genes of CYP: CYP1A1, CYP1A2, CYP2A6, CYP2B6, CYP2C8, CYP2C9, CYP2C19, CYP2D6, CYP2E1, CYP3A4, and CYP3A5 [201]. The diversity was higher in the CYP2 subfamily compared to the CYP1 and CYP3 subfamilies [201]. There were also differences in types and ratios of polymorphism among ethnicities. About 20 to 25% of Caucasians possess a CYP2D6 type with null alleles of *4 (CYP2D6*4, rs3892097) that causes splicing defects and lack of a detectable protein, and thus poor drug metabolism, whereas in Asian, African, and South American peoples, there are almost no mutations of the allele *4 and fewer incidences of symptoms caused by lack of drug metabolism [202]. CYP3A4 and CYP3A5 are involved in most drug metabolism, including tacrolimus and CBD (see Section 5.2 and Section 5.3) and polymorphism in them are known to have large influences on drug metabolism. For example, CYP3A5*3, which is a CYP3A5 with a mutation in intron 3 causing a deficiency (rs776746), lacks a functional CYP3A5 [202,203]. CYP3A5*1 is the functional type of CYP3A5, and it is found much less in Caucasians (5 to 10%) than in African Americans and Africans (more than 60%) [202]. Recent studies have shown that tacrolimus concentration levels in post-organ transplant patients with genotypes of CYP3A5*1/*1 or CYP3A5*1/*3 (homo- or hetero-combination of CYP3A5*1) were significantly lower than these with CYP3A5*3/*3 [204,205]. This suggests that it is important to take into consideration the CYP3A5 type a person possesses when administering drugs. These large differences among ethnic groups in the CYP types that are involved in drug metabolism indicate the importance of determining the types of CYP each patient possesses. 

An important question about these CYP polymorphisms is whether they affect the inhibition of drug metabolism by CBD. So far, there have been no studies that we are aware of that reported differences in the inhibition of drug metabolism by CBD depending on these polymorphisms in CYP [206]. As *1 is the functional type of CYP3A5 and there are large ethnic differences in its expression, it is highly possible that there are differences in the inhibition of drug metabolism by CBD due to the polymorphism in CYP. 

**Figure 10:**
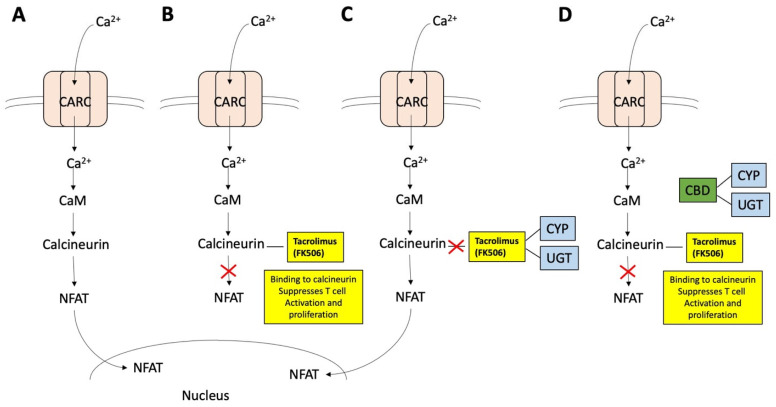
Possible effects of CBD on suppressing tacrolimus metabolism of post-organ transplant patients. (**A**) Post-kidney transplant patients have an elevated immune response caused by surgical injury and a foreign substance, the transplanted kidney. Antigens on antigen-presenting cells, such as major histocompatibility (MHC) molecules, are detected by T cell receptors (TCR), which stimulates calcium influx through CARC, which is sensed by calmodulin (CaM) and activates a calcineurin signaling cascade. Ca^2+^ binds to CaM and calcineurin, and calcineurin dephosphorylate nuclear factor of an activated T cell (NFAT), which translocates to the nucleus and initiates activation of lymphocytes. (**B**) When tacrolimus is present, it binds to calcineurin and blocks the calcineurin signaling cascade. (**C**) If tacrolimus is metabolized by CYP or UGT, calcineurin signaling cascades proceed. (**D**) If CBD is present, it interacts with CYP or UGT, and tacrolimus can block the calcineurin signaling cascade.

Figure 10 illustrates the effects of tacrolimus on suppressing lymphocyte cell activation, how CYPs and UGTs will affect this, and how CBD can inhibit drug metabolism by CYPs and UGTs. Post-organ transplant patients can have an elevated immune response caused by the surgical injury and the transplanted foreign substance, i.e., the transplanted organ. Antigens, or antigens on antigen-presenting cells (APCs), are detected by T cell receptors (TCRs) on T cells, which stimulates initiation of primary signaling cascades (Figure 10A) [2,207,208,209]. Following the primary TCR signaling, multiple distal signaling cascades become activated, including the Ca^2+^-calcineurin-NFAT cascade, which leads to T cell proliferation and differentiation into various subtypes (Figure 10A). When tacrolimus is administered, it interacts with calcineurin, and this can block the Ca^2+^-calcineurin-NFAT cascade and suppress cell proliferation and differentiation (Figure 10B). If tacrolimus is metabolized by CYP or UGT, the T cell proliferation and differentiation process initiated by the antigen proceeds (Figure 10C). Adding CBD will allow it to bind to CYP and UGT, suppress the metabolism of tacrolimus by CYP and UGT, and thus extend the time that tacrolimus functions in suppressing the immune system (Figure 10D).

## 6. Possible Side Effects and Strategies to Overcome

### 6.1. Types of Adverse Events

In the clinical trial reports and reviews listed above (Table 3), some adverse events of CBD were reported. Most often reported adverse events, in general, were gastrointestinal symptoms such as diarrhea, nausea, vomiting, and loss of appetite. Other symptoms reported were drowsiness and dizziness, fatigue, headaches, body weight gain or loss, dry mouth, anxiety, difficulty breathing, mood changes, and rash. Table 4 summarizes some of the reports from clinical trials and review papers. There are five clinical trials in Table 3, and, although the severity is not high, the incidents of adverse events in two of these clinical trials are quite high (both 14/16 in two studies [192,195]). In each of these two studies [192,195], participants were healthy adults, and 5/16 participants and 6/16 participants had to discontinue because of the adverse events, especially based on the elevated levels of liver aminotransferase enzymes, i.e., alanine aminotransferase (ALT) and aspartate aminotransferase (AST) [192,195]. In the third clinical trial [210], in which the participants were patients with symptoms of gastroparesis and delayed gastric emptying, neither ALT nor AST levels were elevated [210]. Although the participants in this clinical trial reported other common adverse events such as diarrhea, nausea, fatigue, and headache, these symptoms were not severe enough to warrant discontinuation from the trial. Importantly, their gastrointestinal symptoms showed significant improvement after taking CBD [210]. 

### 6.2. Strategies to Overcome

As discussed in the previous section, there are adverse events by CBD that would be better to eliminate or decrease. This challenge is not unique to CBD, as many drugs share similar issues. Investigating strategies to minimize adverse events while maintaining the beneficial effects is crucial. Here, we will explore several possibilities.

#### 6.2.1. Co-Administration with Other Phytochemicals

There are many phytochemicals with anti-inflammatory effects [213,214,215,216,217,218] that also have binding affinity with CYP enzymes [141,219,220]. Many of them have different overall profiles of receptors, channels, and enzymes that they interact with. The biggest benefit of using CBD could be its popularity. Based on the long history of using cannabis for medical treatments, CBD can produce ‘meaning responses’ [221], i.e., the placebo effects from expectation that it might work, and this is important. 

The bioavailability of CBD depends on the route it was taken, and it is known that only 6% will be bioavailable when taken through the oral route [222,223]. This low bioavailability necessitates taking a high dose to obtain the expected effects. Unfortunately, CBD turns toxic from a very low concentration, which indicates the importance of a strategy to reduce the dose. The upper limit of CBD described in the prescribing information of Epidiolex is 5 mg/kg body weight per day. For example, a phytochemical compound beta-caryophyllene is a CB2 ligand [224] and activates PPAR gamma and inhibits CYP enzymes [141,225], and has anti-inflammatory effects as well as analgesic effects. Beta-caryophyllene was found to have a toxic effect at doses over 2000 mg/kg body weight per day [141]. This suggests that co-administering with less toxic phytochemicals, such as beta-caryophyllene or others that have a binding affinity with CYP enzymes to reduce the dose of CBD, may produce the same outcome with a lower amount of CBD. Indeed, there are studies testing the combination of CBD and beta-caryophyllene, finding that it produces anti-inflammatory and analgesic effects in a synergistic way [226,227].

The low bioavailability of CBD and gastrointestinal adverse events are quite analogous to the profile of curcumin, which also has low bioavailability and some adverse events but is well-known for its various beneficial effects [228]. Curcumin can be co-formulated with piperine to enhance bioavailability.

Piperine is known to enhance the effects of other phytochemicals due to its inhibitory potency at CYP2C9 and CYP3A4. When it was co-administered with curcumin, it increased the maximum plasma concentration of curcumin by 2000% [18,229]. In another study on co-administering piperine with curcumin, the urinary excretion rate of curcumin 24 h after administration was about 5 times higher than the group who had curcumin alone [230]. An interesting and important study on piperine and curcumin revealed that the effect was multifactorial and it was mediated through the drug–drug interaction (in this case, phytochemical–phytochemical interaction) of binding to CYP3A, which also enhanced the bioavailability through a process of making a chemical complex of curcumin and piperine that enhances the bioavailability of curcumin. Piperine also intercalates with curcumin, most likely through an exchange of protons from curcumin to the carboxyl group of piperine, reforms a hydrogen bond, and establishes a complex with curcumin that enhances transportation. This double-bonded complex improves the bioavailability of curcumin [231]. In other words, these studies indicate that piperine not only directly binds to CYP and UGT enzymes, inhibiting their enzymatic activity, but it also establishes a hydrogen-bonded complex with curcumin and enhances its bioavailability. Whether these same mechanisms take place between CBD and piperine needs to be addressed. There have been studies showing that co-administration of CBD with piperine increased the maximum plasma concentration of CBD [232]. Importantly, in their study [232], they showed not only the effects of co-administration of CBD with piperine, but they also showed the effects of formulation of CBD and of piperine, and showed the efficacy of each formulation as well as the combination of formulated chemical compounds [232]. We will summarize this in the next section.

#### 6.2.2. Enhancing Bioavailability and Reducing the Amount of Administration: Formulation

Currently there are multiple strategies in chemical engineering that exist to enhance the bioavailability of hydrophobic phytochemicals. Table 5 summarizes examples of the chemical agents used as well as the techniques that formulate hydrophobic chemical compounds into complexes with improved bioavailability. 

**Figure 11:**
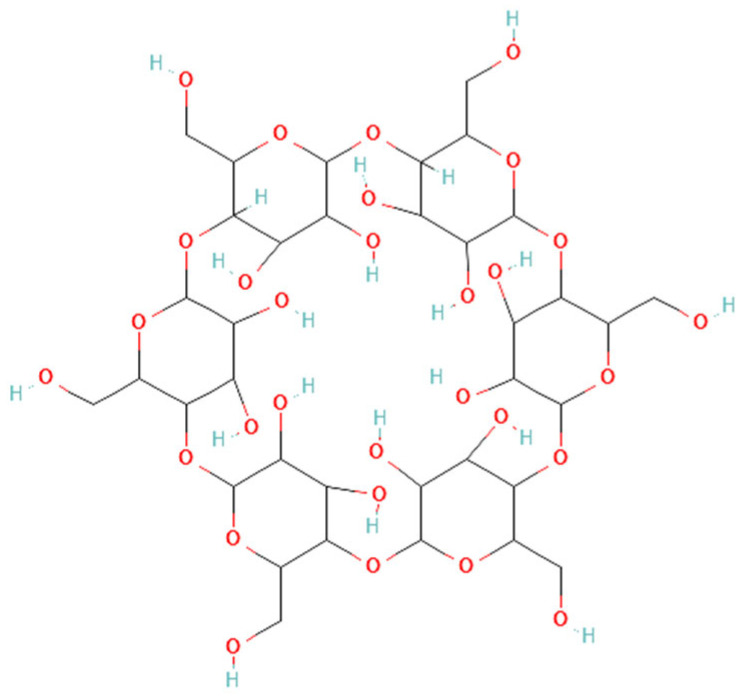
Chemical structure of cyclodextrin. (PubChem CID: 320760; image was retrieved from PubChem: https://pubchem.ncbi.nlm.nih.gov/compound/320760; access date 8 January 2025).

Cyclodextrin inclusion complex: Cyclodextrin (CD) has been known since as early as the 19th century and has been used in the food industry for decades [254,255]. More recently, it has started to be used in drug delivery. CDs have a ring structure (Figure 11) with a hydrophilic outside and a hydrophobic inside, where guest molecules can non-covalently be captured, making a host–guest complex. There are three subtypes, α-CD, β-CD, and γ-CD based on the number of glucoses in the structure, producing differences in the outer diameter, which also affects the inner cavity size [254]. These complexes improve bioavailability by converting hydrophobic molecules into hydrophilic forms and enhancing stability by shielding the guest molecule from enzymatic degradation or oxidation. For example, a study developing CBD-β-CD complexes as anti-inflammatory drugs showed a rapid release of CBD (100% within a minute) and a fast permeation rate of CBD through cultured porcine nasal mucosa, achieving plateau levels of permeation within 10 min. There were almost no cytotoxic influences on cultured human nasal septum tumor epithelial cells (RPMI 2650 cell line) and mouse macrophage-like cells (RAW 264.7 cell line) [233]. Pre-treatment with CBD-β-CD complexes also significantly suppressed pro-inflammatory cytokine secretion in porcine nasal mucosa exposed to the recombinant protein of the spike protein receptor binding domain of SARS-CoV-2 [233]. In another study that generated CBD-cyclodextrin complexes using 2-hydroxypropyl-β-CD (CBD/HP-β-CD), CBD/HP-β-CD complexes promoted cell division and cell migration in cultured human dermal fibroblast cells, suggesting potential for enhancing wound healing [236].

Poly-lactic-co-glycolic acid (PLGA): PLGA has also been known for decades [239]. Its biodegradable nature made it useful for surgical sutures and became used in drug delivery by loading small molecules. It has been used as a controlled drug release agent. In a study that loaded CBD into PLGA nanoparticles (CBD-PLGA-NP), CBD was about 50% released from encapsulation during the first 12 h, and reached to 100% at 96 h (at 37 °C and 5% CO_2_). Internalization in SKOV-3 epithelial ovarian cancer cells occurred 2 h to 4 h after the exposure of cells to CBD-PLGA-NP [237]. In a study using primary chondrocytes from neonate Sprague Dawley rats and exposing them to lipopolysaccharides (LPS), treatment with CBD-PLGA nanoparticles significantly enhanced cell viability and suppressed pro-inflammatory cytokine expression compared to the cells treated with non-formulated CBD [238].

Extracellular vesicles (EVs): EVs are particles secreted from cells, which used to be thought of as debris from cells and recently have been found to have cell-to-cell communication functions [256,257,258]. There are EVs of both mammalian cell origin and plant cell origin, and the EVs of plant cell origin are often called PDEV (plant-derived EV). EVs are broadly categorized into two groups, exosomes and microvesicles [257]. Exosomes are produced by intracellular budding of endosomal membranes and are released by exocytosis, whereas microvesicles are produced by budding of the cell membrane outwards toward the extracellular environment and become released directly into the extracellular environment [257]. Once produced, there are proteins and RNA enclosed in EVs, and these can be delivered to other cells when the secreted EVs attach to the recipient cells and become internalized by endocytosis, phagocytosis, or fusion. The profile of proteins and RNA is affected by the physiological condition of the donator cells, and such changes in the profile are delivered to the recipient cells, and thus cell-to-cell communication takes place. This function to deliver proteins and RNAs has suggested its potential as an agent for drug delivery by encapsulating specific drugs of interest, antigens, vaccines, or genes for, for example, cancer [259,260,261] and other diseases [262] as well as for regeneration and wound healing [263]. Importantly, studies have shown that EVs themselves enhance wound healing and regeneration [264,265], rather than a cargo to deliver drugs, indicating the possibilities of synergetic influences when used as a drug delivery system. These studies suggest the possibility of loading CBD into EVs of mammal origin, such as mesenchymal stem cells (MS-EV) [243], or isolating EVs from *Cannabis sativa*, which contain CBD. Indeed, there are studies isolating EVs from cannabis that contained high amounts of CBD and found that it significantly suppressed the viability of cancer cells [266]. The MS-EV loaded with CBD was also found to have stronger effects than the EV alone and CBD alone on decreasing peripheral neuropathy experimentally induced in mice by administering paclitaxel (8 mg/kg) [243].

Self-emulsifying drug delivery (SEDD, SMEDD, SNEDD): Self-emulsifying drug delivery systems were developed to overcome the problem of low bioavailability in many of the drugs [247,267]. It basically mixes the target drug with oil to encapsulate the drug in the oil drop at the first stage and then with a water-soluble surfactant/co-surfactant with/without cosolvents [267]. Medium-chain triglycerides (C_8_ to C_10_) are better in emulsification, and long-chain triglycerides (>C_10_) are better for drug delivery [267]. As shown in Table 5, the several sub-types are based on the sizes of the formulated SEDD multi-complexes. CBD complexes formulated using this technique showed higher bioavailability compared to free CBD [244] (Table 5).

Flash nanoprecipitation (FNP): The concept of the FNP technique to encapsulate CBD or other drugs of interest is similar to the SEDD method but uses one of two types of chemicals as stabilizers. One is L-α-lecithin, which is a mixture of amphiphilic phospholipids from soybeans (>94% phosphatidylcholine and <2% triglycerides) to form a thin layer on the surface of the nanoparticle, and the other is hydroxypropyl methylcellulose acetate succinate [249]. The “flash” refers to the rapid mixing process, which enables large-scale production of formulated complexes compared to microfluidic device-based methods, which use micrometer-sized laminar flow channels to mix. The FNP method enables the formulation of a large volume quickly.

Others and combinations of multiple techniques: Hybrid methods often combine multiple formulation strategies to improve bioavailability further. Cherniakov et al. (2017a,b) [232,248] used a pro-nanoliposphere technique to formulate cannabinoids and piperine and tested the effects of a combination of multiple chemical compounds with a single administration of them. Interestingly, and importantly, oral administration of formulated CBD-piperine led to a six-fold higher concentration than administration of CBD alone [232]. These findings highlight the potential of combining advanced formulation techniques with the co-administration of phytochemicals like piperine to enhance CBD bioavailability effectively. 

#### 6.2.3. Targeted Drug Delivery

Another possible strategy to reduce the adverse events could be to find a way to limit the targets, i.e., ‘targeted drug delivery’, to certain tissues/organs or types of cells and to delay the release of the drug until they reach the target. This will be possible if a certain protein, such as a biomarker specific to the target cells or biomarkers of cells that are specific to the target tissues/organs, triggers the release of the drug, and until then the drug is encapsulated or biologically inactive. If this strategy is possible, we can, for example, suppress the vitality of only the specific types of immune cells involved in the rejection of transplanted organs without or with less damage to other cells and protect other organs from damage. 

Studies on targeted drug delivery have increased during recent decades, mostly in the study area of oncology. For example, by utilizing biomarkers specific to T cells or specific types of T cells, it is possible to confine the delivery of the drug to these targeted T cells [268,269]. By targeting the delivery to a specific protein involved in the signaling pathways, studies have demonstrated the possibility of enhancing anti-tumor activity [268]. There are multiple clinical trials already conducted or still ongoing, which are testing the effects of drug delivery loaded with CD70, VEGFR2, and others to specifically target tumors or T cells [268]. This strategy has a high possibility of being expanded toward diseases other than cancer and specific conditions such as immunotherapy to post-organ transplant patients with the goal of reducing adverse events.

## 7. Conclusions

CBD can now be obtained over the counter and taken for a variety of purposes, including pain and anxiety. The name that suggests its relationship with cannabis alone can cause “meaning responses”, i.e., placebo effects [221]. This may not sound like a useful factor, but it is, especially because of the extremely well-known name of the plant that contains CBD. The historical background of cannabis as a medicine and recreational compound likely enhances the perceived benefit of CBD. CBD has a number of adverse events and is subject to both pharmacokinetic and pharmacodynamic drug–drug interactions. Likewise, piperine and beta-caryophyllene also have binding affinity with CYP enzymes and anti-inflammatory effects but are less toxic. Yet, many phytochemical biologists know that nobody would expect a major chemical constituent of ‘black pepper’, i.e., piperine and beta-caryophyllene, or of copaiba and rosemary, i.e., beta-caryophyllene, to be effective. Piperine is now included in the supplements of curcumin to enhance its bioavailability, and these supplements are on the market as ‘curcumin’, not as ‘piperine’, although it is at least listed in the ingredients of their labels. To ensure the value of CBD as a drug, it is necessary to identify formulations with enhanced chemical stability that reduce adverse events and necessary dosage by enhancing bioavailability. One option is co-administration of CBD with piperine, as piperine has already shown its effects with curcumin and is on the market as a supplement. In addition, new formulations of CBD and piperine using nanoemulsion or other techniques may further enhance the effects of CBD and further enable dose reduction and avoidance of adverse events. In order to explore the possibility of utilizing CBD in post-organ-transplant patients, it is important to conduct clinical trials and evaluate combination of CBD and tacrolimus to optimize dosing strategies while minimizing adverse effects. Additionally, exploring personalized dosing approaches based on patients’ CYP genotypes would be valuable, as genetic variability significantly impacts CBD metabolism.

## Figures and Tables

**Table 2:** Influences of CBD on the signaling related to immune system, by suppression and activation.

Type of Influences	Reported Influences	References
Suppression/inhibition	Free radical chain reactions were transformed to less active forms by CBD	[78]
	CBD prevented the formation of superoxide radicals and reduced oxidative conditions	[78]
	CBD reduced reactive oxygen species (ROS) by chelating metal ions involved in Fenton reaction. Protects non-enzymatic oxidants from oxidation.	[78]
	CBD suppressed NF-κB through activation of PPARγ: Activation of a nuclear receptor PPARγ, which directly interacted with NF-κB, induced degradation of Rel homology domain of p65 subunit of NF-κB, producing anti-inflammatory effects [78] CBD reduces NF-κB p65 nuclear translocation by suppressing the degradation of interleukin-1 receptor associated kinase 1 (IRAK-1) and reversing inhibitors of κB (IkB). CBD suppresses NF-κB signaling by increasing anti-inflammatory STAT3 phosphorylation and reducing pro-inflammatory STAT1. CBD reduces IL-1β, which is involved in activation of NF-κB [75]. Inhibition of NF-κB increases the expression of nuclear factor 2 associated with erythroid 2 (Nrf2) activators (such as p21 and p62), reducing the inhibitors (such as Keap1, Kelch1, nuclear Bach1), and activated transcription of Nrf2 [168,169] producing antioxidant effects.	[75,76,78,168,169,170]
	Anti-inflammatory effects of CBD may be mediated by its effects on Janus kinase (JAK)-STAT signaling as it suppresses pro-inflammatory STAT1 signaling and regulating IFNβ [75]. CBD down-regulated JAK/STAT pathways, down-regulated IL-6, which is involved in JAK/STAT3 signaling. Also suppressed IL-2 and IFN-γ, which are activators of JAK/STAT signaling. IL-2 is also involved in a signaling pathway that regulates lymphocyte proliferation, PI3K-Akt and MAPK. It also suppresses IL-10, which is also involved in JAK/STAT signaling [66].	[66,75]
	CBD suppresses nucleotide-binding oligomerization domain-like receptor (NLR) signaling pathway and NLR family pyrin domain containing 3 (NLRP3) inflammasome pathway. NLRP3 consists of central nucleotide-binding and oligomerization (NACHT) domain and leucine-rich repeat (LRR) that produces an inflammasome complex. CBD suppresses NLR and suppresses the formation of inflammasome, which suppresses production of caspase 1, IL-1β, and other pro-inflammatory cytokines, interleukin receptors, interferon gamma receptors, mitogen-activated protein kinases, matrix metallopeptidase 3, and so on.	[66,75]
	CBD suppresses IL-1β production.	[75]
	CBD suppresses transcriptional activity of activator protein-1 (AP-1) and nuclear factor of activated T cells (NFAT)	[75]
	CBD inhibits adenylyl cyclase cAMP pathway and produce desensitization in TRPV1 signaling.	[106]
	CBD suppresses FAAH, which suppresses the hydrolysis of anandamide, a partial agonist of CB1 and CB2, and thus increases anandamide levels and leads to indirect activation of CB1/CB2.	[97]
Activation/increase	CBD increases nuclear erythroid 2-related factor (Nrf2), which produces antioxidant effects, and anti-inflammatory effects. Nrf2 controls the expression of genes related to cytoprotective proteins, and activation of Nrf2 produces anti-inflammatory effects.	[78]
	CBD increases glutathione peroxidase and reductase activity and decreases malondialdehyde	[78]
	CBD activates adenosine receptor 2A (A_2A_) and activation of A_2A_ reduces TNFα, vascular cell adhesion molecule (VCAM-1), and prevents reperfusion of oxidative stress in mitochondria. [78] Anti-inflammatory effects by CBD were reversed by A_2A_ antagonist [97]	[78,97]
	CBD activates TRPV1, and increases MDSC, which suppress NK cells, B cells, and T cells by producing arginase, down-regulating the expression of T cell receptor (TCR) components CD3	[72,81]

**Table 3:** Recent clinical trial studies using CBD.

Publication	Target/Goal/Interest	Type of Study	Type of Participants	Main Results
Leino et al. [191]	Goal: Tested the effects of CBD on inhibiting CYP pathway. Interest: Possible use of CBD for post-transplant treatment with tacrolimus	A case report	A participant in a clinical trial study on epilepsy	While receiving CBD, the concentration of tacrolimus increased 3-fold showing significant drug–drug interaction between CBD and tacrolimus. CBD was metabolized by CA3A4 and CYP2C19.
Klein et al. [193]	Interest: epilepsy	Phase 3 trial of clobazam in the treatment of patients with Lennox–Gastaut syndrome	202 participants and comparison with a previous study	Used CBD and anti-CYP drug stiripentol on the level of clobazam, a drug for seizures which becomes metabolized by CYPs, and its active metabolite N-desmethylclobazam, and found they increase the level of N-desmethylclobazam plasma concentration
Patsalos et al. [194]	Interest: safety of using CBD for epilepsy	Review of 6 trials co-administering CBD with anti-seizure drugs either clobazam, stiripentol, valproate, and an inducer of CYP rifampicin, an inhibitor of CYP, itraconazole and fluconazole, and a probe to measure CYP activity midazolam.	Healthy volunteers (*n* = 142) (Phase I trials) or patients with epilepsy (*n* = 55) (Phase 2 trials)	Comparison of several trials’ results showed that co-administration of clobazam and CBD increased exposure to major metabolites of both clobazam and CBD, suggesting the possible need of using reduced doses. Stiripentol with CBD increased exposure to stiripentol. Valproate and CBD did not cause major changes in the pharmacokinetics of either drug but caused a small increase in exposure to CBD metabolites.
Watkins et al. [192]	Interest: to test the liver safety of CBD	Clinical trial phase I	16 healthy adults; gradually increasing the dose up to the largest dose of 1500 mg/day and continued 27 days	14/16 participants experienced adverse events mostly GI symptoms. Elevated serum alanine aminotransferase level higher than upper limit of normal range was observed, suggesting possibility of liver injury.
Thai et al. [195]	Interest: To test the effects of CBD on inhibiting caffeine metabolisms by CYP1A2.	Clinical trial Phase I	Healthy adults (*n* = 16); one dose of caffeine (200 mg) on day 1 and again on day 26 (200 mg). CBD was taken daily for 27 days, gradually increasing from 250 mg to 750 mg/day	CYP1A2 mediated metabolite of caffeine, paraxanthine, was measured on day 1 (pre-dose of CBD) and day 26 (steady state of CBD). Caffeine level was higher by 15% when it was administered at steady state. Paraxanthine was also higher when caffeine was administered at the steady state of CBD. No unexpected adverse events but 14/16 participants reported adverse events. Diarrhea was observed most often (8/16), and 6 participants discontinued because of adverse events. CBD was found to inhibit CYP1A2.
Peng et al. [63]	Interest: Summarizes clinical trials on mechanisms of action. Summarizes the multiple receptor types that CBD activates/inactivates, and compares studies and clinical trials	A review covering clinical trials and mechanisms		Summarizes clinical trials on psychotic, anxiety, epilepsy, insomnia, blood pressure, diabetes, pain, and cancer.
Graham et al. [196]	Interest: to obtain information on how CBD will interact with drugs used and to provide general guidance about the dose range.	A review covering clinical trials		Summarizes a table with a list of clinical trials using various drugs co-administered with CBD with their concentrations and results, including adverse events.
Bansal et al. [197]	Interest: To determine the influences of CBD and THC on CYP.	Clinical trial	Healthy adults (*n* = 18);	Used brownies with/without CBD and/or THC, and consumed a CYP cocktail containing CYP1A2, CYP2C9m CYP2C19, CYP2D6, and CYP3A. THC alone did not inhibit CYP activity but CBD + THC did, suggesting the possible use of CBD for co-administering with drugs which will interact with CYPs.
Herdegen and Cascorbi [198]	Interest: To determine the influences of THC and CBD when they are prescribed to patients taking other drugs.	A review covering other clinical trials		Drug interactions may happen when the doses are above 30 mg/day THC and 300 mg/day CBD; low and moderate doses will probably not affect. The paper also summarizes the types of adverse events and reported percentages of each type.
Luz-Veiga et al. [199]	Interest: To summarize receptors that CBD interacts with and therapeutic application.	A review covering clinical trials		Provides lists of clinical trials using CBD, separating them according to their goals, i.e., neurologic conditions, autoimmune diseases, and cancer.
So et al. [200]	Interest: To determine whether steady-state CBD exposure affects tacrolimus pharmacokinetics in healthy participants	Clinical trial	Healthy (*n* = 12)	Participants received CBD and tacrolimus at naïve stage and after two weeks taking CBD (steady-state). Tacrolimus concentration in blood was higher in the participants at the steady-state of CBD, suggesting the possible effects of CBD on inhibition of CYP.

**Table 4:** Reported adverse events of CBD in clinical studies.

Authors and Year	Subject F: Female, M: Male	Types of Adverse Events (Y: Yes, N: No, U: Unknown)							Dose, Route of Administration, and Duration	Notes	References
		Mood, Anxiety	GI: Nausea, Diarrhea, Appetite	Drowsiness/ Dizziness	Fatigue Sleepiness	Breathing	Skin	Other			
Iffland and Grotenherman	Review	U	Y: diarrhea, reduced appetite	U	Y: Tiredness	U	U	Weight loss, liver damage			[48]
Huestis et al.	Review	U	Y: diarrhea, vomiting, loss of appetite, abdominal discomfort, increased appetite	Y: somnolence, drowsiness, dizziness	Y: fatigue	U	Y: rash	Hepatic abnormalities, headache, weight gain/loss, elevated liver aminotransferase enzymes, fever, upper respiratory tract infection, sexual dysfunction			[211]
Watkins et al.	Healthy adults, *n* = 16 (10F, 6M), median of age 29, >80% white	U	Y 8 (50%): diarrhea, 5 (31%): abdominal discomfort	U	U	U	U	6 participants had ALT values over ULN	Increasing from 200 mg once/day, 250 mg twice/day, (500 mg + 250 mg)/day, 500 mg twice/day, (750 + 500)/day, 750 mg twice/day for 27 days, oral	14/16 reported adverse events; 5/14 discontinued because of adverse events, specifically, the ALT higher than ULN	[192]
Thai et al.	Healthy adults, *n* = 16 (10F, 6M), 18–60 years old, >80% white	U	Y 8 (50%): diarrhea	U	U	U	U	N = 1, high blood ALT	Increasing from 250 mg once/day to 750 mg twice/day for 27 days, oral	14/16 reported adverse events; 6/14 discontinued because of adverse events	[195]
Herdegen and Cascorbi	Review	Irritability	Reduction in appetite, diarrhea	Y	Y	U	U	Dry mouth, aggression			[198]
Nguyen et al.	Adult (average age 43)	Anxiety (>23%), mood change (>16%)	diarrhea (>7%), nausea (>4%), appetite change (>15%)	drowsiness (>11%), dizziness (>9%),	U	difficulty breathing (>2%)	rash (>3%), dry moth (>18%),	liver problems (>3%)	Varied as the study was conducted as a survey; not necessarily pure CBD and the adverse events may include those caused by other chemical compounds	Varied as the study was conducted as a survey	[212]
Zheng et al.	Patients with non-surgical gastroparesis with delayed gastric emptying of solids, *n* = 44 (*n* = 21 CBD, *n* = 23 placebo), *n* = 19/21 F, >90% white	U	Diarrhea (12/21; of the participants of placebo group, which received excipient 2/22 had diarrhea), nausea (3/21; placebo group nausea, 4/22); only diarrhea was significantly different	U	Fatigue (4/21)	U	U	The score of gastrointestinal symptoms of CBD group measured by GCSI-DD was significantly improved in finishing meals, vomiting episodes, and perceived symptoms.	4 weeks, starting from 2.5 mg/kg/day to 20 mg/kg/day, oral, split in twice daily; 4/21 participants could not tolerate full dose.	ALT and AST level all normal; 5/44 could not tolerate full dose	[210]
Naya et al.	Review	Y	Y	Y	Y	U	Y	Headache, pain, suicidal thoughts, weight loss, elevated ALT/AST, seizures, fever, upper respiratory tract infection			[87]
So et al.	Healthy adults	U	Y	N	Y	U	y	Poor sleep, euphoria, vivid dream			[200]

ULN: upper limit of normal; ALT: alanine aminotransferase; AST: aspartate aminotransferase; GCSI-DD: gastroparesis Cardinal Symptoms Index Daily Diary.

**Table 5:** Types of chemical agents and formulation techniques used.

		Techniques Used and Effects	References
Agents			
Cyclodextrin inclusion complex		Water-soluble nasal spray of CBD developed with β-cyclodextrin-CBD complex (CBD-β-CD) was tested effective in ex vivo nasal mucosa immune cells [233].	[233,234,235,236]
Poly-lactic-co-glycolic acid (PLGA)		PLGA loaded with CBD showed controlled release of CBD for 96 h in ovarian cancer cells [237]. CBD-PLGA showed higher bioavailability of CBD and significantly suppressed inflammatory cytokines [238].	[237,238,239]
Archaeosomes		Archaeosomes are liposomes made with ether lipids from Archaeobacteria [240]. A comparison of two types of formulated CBD, archaeosomes-CBD and soybean lecithin-derived liposome-CBD, revealed 6 times higher uptake of archaeosome-CBD compared to lecithin-derived liposome-CBD in in vitro assays using Caco-2 cells [241].	[240,241]
Extracellular vesicles (EV)		Mammalian-derived EV and plant-derived EV can be carriers for drug delivery and EV themselves are found to have anti-inflammatory effects, anti-cancer effects, and to improve wound healing and regeneration.	[242,243]
Techniques			
Self-Emulsifying Drug Delivery System (SEDDS)	SEDDS (>300 nm)	Single dose of SEDDS-CBD (25 mg CBD) produced 4.4 times higher C_max_ and faster T_max_ (1.0 h compared to 3.0 h in control CBD). Control CBD showed sex differences in bioavailability (F > M), which diminished in SEDDS-CBD [244]	[244,245,246]
	Micro-emulsifying (SMEDDS) (<250 nm)	Names of SEDDS, SMEDDS, SNEDDS indicate the sizes of the produced complex.	[247]
	Nano-emulsifying (SNEDDS) (<100 nm)	Pro-liponanospheres (or pro-nanolipospheres) of CBD with piperine produced 4 times higher C_max_ and 2.2 times higher AUC in healthy human participants [248] and 6 times higher AUC in a rat model [232].	[232,248]
Flash Nanoprecipitation		Uses rapid mixing method at large volume compared to methods using microfluidics. In addition, utilizes lecithin or hydroxypropyl methylcellulose acetate succinate as stabilizer and iron oxide (Fe_3_O_4_) to add density, which improves separation of formulated CBD from free CBD [249]	[249,250,251]
UltraShear nanoemulsion		Utilizes high pressure (30,000 psi) for homogenization. Formulated CBD (20 mg/mL) was administered to rats (5 mg/animal by gavage), which produced average relative bioavailability of 18.6% at 6 h post-administration and 25.4% at 24 h post-administration	[252,253]

C_max_: maximum concentration in blood, T_max_: time until the concentration reaches maximum.
